# Editorial: New perspectives and innovative techniques in contemporary spine surgery—volume II

**DOI:** 10.3389/fsurg.2026.1925692

**Published:** 2026-08-05

**Authors:** Luca Ambrosio, Gianluca Vadalà, Fabrizio Russo, Daisuke Sakai, Vincenzo Denaro

**Affiliations:** 1 Operative Research Unit of Orthopaedic and Trauma Surgery, Fondazione Policlinico Universitario Campus Bio-Medico, Rome, Italy; 2 Research Unit of Orthopaedic and Trauma Surgery, Departmental Faculty of Medicine, Surgery and Dentistry, Università Campus Bio-Medico di Roma, Rome, Italy; 3 Department of Orthopedic Surgery, Surgical Science, School of Medicine, Tokai University, Isehara, Japan; 4 Center for Musculoskeletal Innovative Research and Advancement (C-MiRA), Tokai University Graduate School, Isehara, Japan

**Keywords:** artificial intelligence, Endoscopic spine surgery, minimally invasive spine surgery, navigation, Spinal Cord, Spine

The continuous evolution of spine surgery is increasingly driven by the integration of technological innovations, refined surgical strategies, biomechanical research, and patient-centered outcome assessments. While the first volume of this Research Topic highlighted the potential of emerging technologies to reshape contemporary spinal care, this second collection further expands the discussion by illustrating how innovation may improve surgical precision, procedural efficiency, safety, clinical decision-making, and postoperative recovery.

Importantly, innovation in spine surgery should not be intended merely as the adoption of new devices or techniques. Rather, it should represent a multidisciplinary process that integrates engineering, imaging, surgical expertise, clinical research, biological understanding, and patient-reported outcomes. The 19 contributions in this Research Topic reflect this broader perspective, spanning minimally invasive and endoscopic surgery, navigation and three-dimensional (3D) printing, deformity correction, implant design, perioperative safety, outcome measurement, and translational research.

Several articles addressed the role of novel technologies in improving the accuracy of surgical planning, execution, and instrumentation in anatomically demanding upper cervical procedures. For example, Ruiqiang et al. compared navigation-assisted and patient-specific 3D-printed guidance techniques for C2 pedicle screw placement in patients with Hangman's fractures, including cases with high-riding vertebral artery variants. Both techniques achieved excellent accuracy and avoided neurovascular complications; however, the 3D-printed templates were associated with shorter screw insertion time and lower intraoperative blood loss. Similarly, Zhang et al. investigated the application of a 3D-printed navigation template during transforaminal lumbar interbody fusion (TLIF) for lumbar spinal stenosis (LSS). Their study showed reduced operative time, blood loss, and radiation exposure, together with improved pedicle screw accuracy compared with conventional freehand placement. Qi et al. presented a technical note describing the use of bilateral crossing C2 laminar screws combined with a dedicated screw placement assistant tool. In their preliminary series, this technique allowed for accurate bicortical laminar screw placement without vertebral artery injury, spinal canal breach, implant failure, or need for reoperation. In a related contribution, Muthu et al. reported using 3D-computed tomography (CT) navigation in the management of sudden-onset myelopathy caused by os odontoideum and atlantoaxial instability. Their case highlights the potential role of navigation in enhancing confidence and accuracy during complex craniovertebral junction instrumentation.

Beyond hardware placement, imaging-derived parameters may also improve surgical decision-making. Cui et al. evaluated the combined use of the modified K-line and modified spinal cord line to select laminoplasty segments in patients with multilevel cervical spondylotic myelopathy. Their findings suggest that the presence of both positive indicators on sagittal magnetic resonance imaging (MRI) can help identify patients who can expect adequate posterior spinal cord shift and favorable neurological recovery following laminoplasty.

Minimally invasive and endoscopic procedures remain a central theme of contemporary spine surgery. Chen et al. compared percutaneous endoscopic transforaminal discectomy and unilateral biportal endoscopic (UBE) discectomy for L4–L5 disc herniation. Both techniques achieved substantial improvements in pain and disability, although their respective strengths appeared to differ according to herniation morphology. Percutaneous endoscopic transforaminal discectomy was associated with reduced blood loss and a shorter operative time, whereas UBE discectomy appeared particularly suitable for migrated disc herniations due to the shorter fluoroscopy time and easier portal placement. The comparative role of different endoscopic approaches was further explored by Yu et al. in a systematic review and meta-analysis comparing UBE and uniportal endoscopy for LSS. The authors found comparable outcomes in terms of postoperative pain relief, functional recovery, blood loss, hospital stay, and complication rates, while UBE was associated with shorter surgical duration. Pan et al. investigated a more specific technical aspect of endoscopic posterior lumbar interbody fusion (PLIF) by comparing guidewire placement before and after decompression in patients with unstable LSS. Guidewire placement before decompression was associated with reduced operative time, fewer fluoroscopic acquisitions, and better pedicle screw accuracy, while clinical improvement, fusion rates, and complication rates were similar between groups. The potential expansion of endoscopy to less common indications was illustrated by Li et al., who reported on UBE decompression for symptomatic spinal epidural lipomatosis. Their case demonstrated satisfactory neurological recovery, limited blood loss, a short hospital stay, and sustained improvement at follow-up, suggesting that endoscopic approaches may represent a feasible alternative to open surgery in selected patients.

Innovation is also relevant to spinal deformity surgery, where optimizing correction while minimizing invasiveness, morbidity, and implant burden remains crucial. Li et al. compared a modified halo-pelvic traction technique combined with pedicle subtraction osteotomy to conventional halo-pelvic traction in patients with severe adolescent idiopathic scoliosis (AIS). The modified protocol achieved greater correction during traction, improved pulmonary function, and a lower overall complication rate while maintaining satisfactory correction at the 1-year follow-up. Zheng et al., in a systematic review and meta-analysis including 1,762 patients, evaluated low-density versus high-density pedicle screw constructs in AIS. Low-density constructs were associated with lower blood loss, shorter operative time, and reduced implant-related costs, without significant differences in radiographic correction, revision rates, or complications. These findings support the concept that more extensive instrumentation is not necessarily associated with better outcomes.

The importance of biomechanical optimization in reconstructive spine surgery was addressed by Gu et al., who compared artificial vertebral bodies and titanium mesh cages following total en bloc spondylectomy in the thoracolumbar spine. Finite element analysis showed a more favorable stress distribution with artificial vertebral bodies, while the clinical cohort demonstrated lower implant subsidence, reduced loss of vertebral height, and improved maintenance of segmental alignment. These findings highlight the importance of implant design in reducing mechanical failure and improving long-term reconstruction outcomes after complex oncological spine surgery. Luke et al. contributed an important engineering perspective by validating a modular benchtop device capable of replicating the impact energy absorbed during cadaveric TLIF. By reproducing selected waveform characteristics observed during cage impaction, this model may offer a reproducible and efficient platform for evaluating the design of interbody fusion devices, material behavior, and implant failure mechanisms. These preclinical models could accelerate device testing while reducing reliance on cadaveric experimentation.

Innovation also includes improving the way clinical research is designed, conducted, and interpreted. Schiegg et al. used Protocol AI to analyze historical delays and early termination in spinal fusion trials. Their findings showed that approximately 25% of trials were terminated prematurely and that completed trials experienced a mean delay of more than 10 months, with recruitment difficulties representing a significant challenge. These results underline the need for more efficient trial design, realistic recruitment strategies, and data-driven approaches to improve the translation of promising technologies into clinically meaningful evidence.

The evaluation of surgical success should also extend beyond conventional radiographic and clinician-reported outcomes. Ambrosio et al. presented the study protocol for the Forgotten Spine Score, a novel patient-centered outcome measure specifically designed for patients undergoing lumbar spine fusion. Inspired by the Forgotten Joint Score, the 18-item questionnaire aims to assess the extent to which patients can “forget” the operated spinal segment during everyday activities. The proposed validation in Italian and Japanese populations may provide a valuable tool to complement established disability measures and better capture patients' perceptions of recovery.

Several contributions focused on the prevention and management of complications, further emphasizing that innovations should improve not only treatment efficacy but also perioperative safety. Su et al. investigated the association between visceral fat area and surgical site infection after posterior thoracolumbar surgery. In their large retrospective cohort, visceral fat area was a stronger predictor of infection than body mass index (BMI), and visceral fat obesity was associated with a substantially higher infection rate. These findings support the potential value of body composition assessment in preoperative risk stratification. Korovessis et al. performed a systematic review and meta-analysis to compare conservative treatment with vertebral augmentation for osteoporotic vertebral compression fractures. Their analysis of randomized studies suggested a lower incidence of adjacent vertebral fractures after vertebroplasty or kyphoplasty compared with conservative treatment, whereas retrospective studies showed no significant difference. This discrepancy highlights the persisting uncertainty surrounding adjacent-level fracture risk and the need for adequately powered prospective studies that account for baseline osteoporosis severity, vertebral alignment, cement-related factors, and patient selection.

Finally, the collection also explores emerging biological and physiological dimensions of spinal disorders. Jeyaraman et al. reviewed the implications of gut microbiome alterations after spinal cord injury, focusing on their relationship with systemic inflammation, immune dysregulation, intestinal permeability, chronic pain, infections, and neuroplasticity. Their work identifies the gut-spinal cord axis as a promising area for future translational research and suggests that microbiome-targeted interventions may eventually complement current rehabilitation and neuroprotective strategies. Ren et al. contributed a preclinical study using laser speckle contrast imaging to monitor spinal cord hemodynamics during pedicle subtraction osteotomy in rabbits. The authors demonstrated dynamic changes in spinal cord perfusion and posterior spinal artery diameter during different surgical phases, supporting the potential of real-time optical monitoring as a future adjunct for spinal cord protection during complex deformity correction.

Collectively, the studies included in this Research Topic demonstrate that the future of spine surgery depends on the convergence of technical refinement, individualized planning, robust clinical evidence, and meaningful patient-centered assessment. Navigation, three-dimensional printing, endoscopy, advanced implant design, biomechanical modeling, artificial intelligence, and biological research all have the potential to improve spinal care. However, the translation of these innovations into routine practice requires rigorous validation, reproducible reporting, adequate training, long-term follow-up, and careful attention to clinical relevance.

We sincerely thank all the authors, reviewers, editors, and readers who contributed to this second volume. Their work provides valuable insights into the current direction of spine surgery and reinforces the importance of multidisciplinary collaboration in shaping a safer, more precise, and more patient-centered future for the field.

